# Pharmacologic Modulation of Hand Pain in Osteoarthritis: A Double-Blind Placebo-Controlled Functional Magnetic Resonance Imaging Study Using Naproxen

**DOI:** 10.1002/art.38987

**Published:** 2015-02-25

**Authors:** Duncan Sanders, Kristina Krause, Jonathan O'Muircheartaigh, Michael A Thacker, John P Huggins, William Vennart, Nathalie J Massat, Ernest Choy, Steven C R Williams, Matthew A Howard

**Affiliations:** 1 King's College LondonLondon, UKUniversity of Sydney at Royal North Shore HospitalSydney, New South Wales, Australia; 2 King's College LondonLondon, UKJustus Liebig University GiessenGiessen, Germany; 3 King's College LondonLondon, UK; 4 King's College London and Guys and St. Thomas' NHS Foundation TrustLondon, UK; 5 Pfizer Global Research and DevelopmentSandwich, UK; 6 Pfizer Global Research and Development, Sandwich, UK, and Queen Mary University of LondonLondon, UK; 7 Cardiff UniversityCardiff, UK

## Abstract

**Objective:**

In an attempt to shed light on management of chronic pain conditions, there has long been a desire to complement behavioral measures of pain perception with measures of underlying brain mechanisms. Using functional magnetic resonance imaging (fMRI), we undertook this study to investigate changes in brain activity following the administration of naproxen or placebo in patients with pain related to osteoarthritis (OA) of the carpometacarpal (CMC) joint.

**Methods:**

A placebo-controlled, double-blind, 2-period crossover study was performed in 19 individuals with painful OA of the CMC joint of the right hand. Following placebo or naproxen treatment periods, a functionally relevant task was performed, and behavioral measures of the pain experience were collected in identical fMRI examinations. Voxelwise and a priori region of interest analyses were performed to detect between-period differences in brain activity.

**Results:**

Significant reductions in brain activity following treatment with naproxen, compared to placebo, were observed in brain regions commonly associated with pain perception, including the bilateral primary somatosensory cortex, thalamus, and amygdala. Significant relationships between changes in perceived pain intensity and changes in brain activity were also observed in brain regions previously associated with pain intensity.

**Conclusion:**

This study demonstrates the sensitivity of fMRI to detect the mechanisms underlying treatments of known efficacy. The data illustrate the enticing potential of fMRI as an adjunct to self-report for detecting early signals of efficacy of novel therapies, both pharmacologic and nonpharmacologic, in small numbers of individuals with persistent pain.

Osteoarthritis (OA) is a major cause of long-term disability (1). Traditionally characterized by structural degradation of one or more synovial joints, OA is typically associated with pain, swelling, and stiffness (2). Reduced manual dexterity and inability to perform tasks due to symptoms affect quality of life and ability to function independently (3). Levels of pain and disability experienced by patients often do not correspond to the level of tissue damage observed in the affected joints (4), suggesting involvement of central nervous system mechanisms in the generation of OA-related pain (5). Given the prevalence of OA and an increasingly aging population, there is a pressing need for more effective treatments (6,7). Many commonly prescribed medications are ill suited for long-term use due to side effects including tolerance, dependency, and gastrointestinal complications (8,9). While pain is always a personal experience and subject to multiple psychosocial factors, there has long been a desire to complement subjective reports with reliable markers of pain perception and treatment effectiveness (10,11).

Neuroimaging methodologies offer the potential to meet these desires (12). Functional magnetic resonance imaging (fMRI) provides a safe, noninvasive, repeatable indirect measure of neural activity suitable for use in crossover trial designs (12). Reports have suggested that fMRI could demonstrate analgesic effects in populations of patients with clinical pain, adding value to conventional behavioral end points and facilitating decision-making early in drug development, reducing risk, development times, and cost (12). Experimental fMRI studies have provided useful insights into the neural mechanisms of drug action (for review, see ref.(13)). In order to fulfill this potential, fMRI must depict the neural correlates of the response to analgesics in patients experiencing persistent pain. Preliminary studies in OA have shown promise in establishing neural correlates of response to analgesics (14,15), but to date these studies have been underpowered or lacked appropriate placebo control. In this study we investigated the potential of fMRI as a “fit-for-purpose” measurement technique in development of analgesics for persistent pain.

We sought to determine the ability of fMRI to detect changes in brain functioning following a 1-week course of naproxen in individuals with OA of the first carpometacarpal (CMC) joint. Naproxen is an established, effective nonsteroidal antiinflammatory drug (NSAID) labeled for treatment of OA-related symptoms in the UK (http://www.emc.medicines.org.uk). We aimed to detect naproxen-induced changes in brain activity using a functionally relevant task that evoked pain. We hypothesized that task-evoked brain activity would be reduced following administration of naproxen, compared to placebo. In particular, we hypothesized that a network of brain regions previously associated with pain perception would display significant reductions in activity following naproxen administration, during the task load that induced the highest rating of pain intensity.

## Patients and Methods

### Design

This study was a placebo-controlled, double-blind, 2-period crossover design undertaken in participants with OA of the first CMC joint of the right hand.

### Ethics

Ethical approval was obtained from The Joint South London and Maudsley and The Institute of Psychiatry NHS Research Ethics Committee (reference no. 10/H0807/10). Written informed consent was obtained from all participants.

### Participants

Participants were recruited from multiple sources, including national radio, local and national print magazines, a university e-mail circular, and referrals from hospital rheumatology and physiotherapy departments. All participants visited on 4 occasions: screening, familiarization, and 2 scanning sessions (1 following each treatment period). All had a confirmed diagnosis of OA of the first CMC joint of the right hand according to American College of Rheumatology criteria (16) and were right-hand dominant with a background pain intensity score of ≥4 on an 11-point (0–10) numerical rating scale (NRS) at screening or randomization. Normal exclusion criteria for MRI applied (e.g., height and weight criteria, presence of internal metal, claustrophobia). Several other major exclusion criteria were also applied, including severe pain elsewhere in the body that might impair the assessment of OA-related pain, history of other severe acute or chronic medical or psychiatric conditions, previous nonresponse to naproxen for pain relief, increased risk of adverse reactions to NSAIDs, inability to conform to lifestyle guidelines, and use of prohibited medications (including analgesics other than NSAIDs, paracetamol, or codeine) prior to and during study participation. Detailed inclusion/exclusion criteria as well as screening, blinding, and randomization information are provided in Supplementary Materials, available on the *Arthritis & Rheumatology* web site at http://onlinelibrary.wiley.com/doi/10.1002/art.38987/abstract.

**Figure 1:**
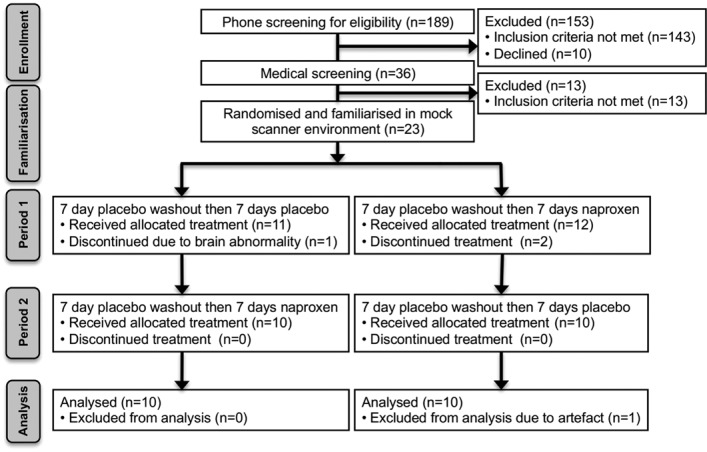
Overview of study procedures and experimental design.

A total of 189 participants (131 women, 58 men) completed a preliminary telephone interview; 36 met the inclusion criteria for screening, and 23 were randomized into the study. Four participants were excluded after randomization, due to radiologic abnormalities, drug-related adverse reactions, and an MRI hardware failure. Nineteen participants (18 women, 1 man) ages 50–80 years (mean ± SD 60.72 ± 6.44 years) were included in the final analysis set. All participants who completed the study reported compliance with the dosing regimen (Figure 1). (Approximately 30% of all prospects for the study were male, but only 18% were screened. Exclusions in men were predominantly health related, for example, severe pain elsewhere in the body, other types of hand pain [e.g., repetitive strain disorder/trigger finger], contraindicating medical/surgical histories, head trauma, and lifestyle guideline exclusions.)

### Procedure

All participants completed psychometric and pain-related questionnaires at each session, including the short form McGill Pain Questionnaire (MPQ) (17), the Beck Depression Inventory II (BDI-II) (18), the Spielberger State-Trait Anxiety Inventory (STAI) (19), and the Patient-Rated Wrist and Hand Evaluation (PRWHE) (20).

### Screening

At the initial session patients underwent a medical and comprehensive blood screening, and medication history was ascertained. Urine drug screen and alcohol breath tests were administered at every session to ensure no confounding drug or alcohol use. Participants also underwent a clinical hand examination by a specialist physiotherapist.

### Familiarization

During the familiarization and measurement sessions, each participant underwent a “mock scan” in a simulated MRI environment designed to reduce feelings of anxiety and claustrophobia. During the mock scan, participants received training on the task they would perform during actual fMRI sessions, including a complete “trial run” and participant-specific calibration of the task.

### Medication dosing

All participants underwent down-titration of existing analgesic medications over a period of up to 28 days. Following down-titration, all participants received a 14-day paper diary in which to record perceived pain intensity every morning and evening using an 11-point (0–10) NRS and to record any observations or changes relating to their hands or general health. In the first 7 days, all participants were administered placebo. In the remaining 7 days, participants were randomly administered either naproxen (500 mg twice a day) or matched placebo. Following the first fMRI session, all participants received a further 7 days of placebo and then either naproxen or placebo (crossed over from period 1) as their second period of dosing. Naproxen has well-established pharmacokinetics, with peak plasma concentration 2–4 hours after ingestion and a plasma half-life of 12–15 hours (http://www.emc.medicines.org.uk).

### Functional MRI scanning sessions and followup

The fMRI task was designed to evoke OA-related pain (see below). After completion of the scanning session, medication containers and pain diaries were given for the second dosing period. The final fMRI scanning session was identical to the first, except that no further treatment was dispensed. Six additional telephone checks were performed during the study to ensure well-being and compliance, and a followup telephone interview was conducted ∼2 weeks after the final fMRI session.

### Task

The task involved squeezing a key-shaped pressure device between the right thumb and index finger (lateral pinch) of the affected hand, designed to mimic everyday activities difficult for individuals with OA hand pain, such as gripping or handling small objects (3). The lateral pinch device was an MRI-compatible pressure transducer, to which participants applied varying levels of pressure. The device was calibrated for each participant during the familiarization session to determine the maximum voluntary contraction (MVC) for each individual, measured in kilopascals. MVC was defined as the average of 3 lateral pinches at each participant's maximum effort. During the 23-minute event-related fMRI task, participants were cued to perform the lateral pinch (squeeze) at each of 3 different levels (10%, 40%, and 70%) of their MVC. The target force to be applied and visual feedback of the participant's own applied force were provided via a visual target and moving vertical line on a custom computerized scale projected onto a screen located at the participant's feet.

In each trial, participants were instructed to squeeze the device until they reached the displayed target and then perform an isometric hold for 1 second. The time required to reach the desired effort was recorded to account for variability in motor task duration. Each trial cycle lasted for 24 seconds. Stimulus onsets were jittered within the first 6 seconds of each trial. In addition, 15 (5 per level of MVC) computerized, horizontal visual analog scale (VAS) assessments of pain intensity were presented pseudorandomly throughout the experiment, during which participants rated their perceived pain intensity during the last-performed squeeze. Participants performed VAS assessments using a joystick in their left hand, anchored at the left with “no pain” and at the right with “worst pain imaginable.” The VAS was displayed for 9 seconds to allow adequate response time, followed by a 12-second intertrial interval. In total, 60 trials were presented in each experimental run—15 per percentage of MVC and 15 VAS ratings. Illustrations of the pinch device, target display, and computerized VAS are provided in Supplementary Figure 1, available on the *Arthritis & Rheumatology* web site at http://onlinelibrary.wiley.com/doi/10.1002/art.38987/abstract.

### Behavioral data analysis

Pain diary and psychometric data were analyzed using SPSS version 19. Differences between treatments on the PRWHE, STAI, BDI-II, and short form MPQ were assessed using paired *t*-tests.

### In-scanner behavioral analyses

In-scanner VAS ratings were analyzed using repeated-measures analysis of variance, with MVC level (10%/40%/70%) and treatment (naproxen/placebo) as factors.

### MRI data acquisition

MRI data were acquired on a 3T Signa HDx whole-body scanner (General Electric) fitted with an 8-channel, phased-array receive-only head coil. A total of 465 whole-brain T2*-weighted interleaved axial volumes were acquired using echo-planar imaging. A high-resolution T1-weighted 3-dimensional (3-D) spoiled gradient-recalled acquisition in the steady state (SPGR) sequence was also acquired for interparticipant registration. Finally, we sought to investigate whether treatment-related variability in the blood oxygen level–dependent (BOLD) response (21) might be explained by its relationship to underlying changes in global cerebral blood flow (CBF)—a possibility given the modulatory effect of naproxen on prostaglandin production (22), which in turn modulates vascular tone, blood pressure, and global CBF. We assessed global CBF using a pulsed continuous arterial spin–labeled (pCASL) perfusion MRI sequence acquired immediately prior to fMRI at each session (23,24). Acquisition order and parameters were identical in both scanning sessions. Full details regarding all sequence parameters are provided in Supplementary Materials, available on the *Arthritis & Rheumatology* web site at http://onlinelibrary.wiley.com/doi/10.1002/art.38987/abstract.

### Functional MRI data preprocessing

Preprocessing was conducted using Statistical Parametric Mapping software (SPM8) (http://www.fil.ion.ucl.ac.uk/spm/software/spm8/). Preprocessing included motion correction, coregistration of the 3-D SPGR image to the mean functional image, segmentation, and normalization to Montreal Neurological Institute (MNI152) template space. Spatial smoothing using a full-width half-maximum Gaussian kernel of 8 mm, interleaved slice timing correction, and high-pass temporal filtering (100 seconds) were performed using FMRI Expert Analysis Tool (FEAT) version 5.98 (http://www.fmrib.ox.ac.uk/fsl).

### Functional MRI statistical analysis

Statistical analysis of fMRI data was carried out using FEAT. A generalized linear model for each participant and session was constructed to perform the voxelwise time series statistical analysis using FSL-FILM. Event timings at 10%, 40%, and 70% MVC were convolved with a double-gamma hemodynamic response function and its temporal derivative. Temporal autocorrelation correction was used (25), and estimated motion parameters were included as confound regressors. Individual participant first-level contrast of parameter estimates (COPE) images, under both naproxen and placebo conditions, were derived for 10%, 40%, and 70% MVC. An intermediate second-level fixed-effects analysis was performed to average the 10%, 40%, and 70% MVC COPE images to take forward for higher-level analysis.

### Higher-level group fMRI statistical analysis

Higher-level analysis was carried out using FSL-FLAME stage 1 with robust outlier detection (26). A whole-brain mixed-effects analysis was performed, using individual participants' COPE images derived from the second level as inputs, to compute voxelwise paired *t*-tests comparing differences in evoked brain activity between naproxen and placebo conditions. An additional confound regressor for medication administration order was included in the model. Z (Gaussianized T/F) statistic images were thresholded using clusters determined by Z >2.3 and a (Gaussian random field–corrected) cluster significance threshold of *P* = 0.05 (27).

### Global CBF analysis

A measure of global CBF in each session was defined as the mean of all gray matter voxels within the 3-D pCASL volume. Differences in global CBF between naproxen and placebo sessions were assessed using a paired *t*-test in SPSS version 19. The significance threshold was set at *P* < 0.05.

### Region of interest (ROI) analyses

Anatomic ROIs in MNI space were derived from Harvard-Oxford Cortical/Subcortical and Juelich Histological probabilistic atlases available within FSL. Based on a priori information regarding brain activation related to pain (28), ROIs were created for the thalamus, primary and secondary somatosensory cortices, hippocampal formation, anterior and posterior insula, anterior and posterior cingulate cortices, and amygdala in both hemispheres. Probabilistic ROIs were thresholded to include voxels more than 20% likely to represent their indicated anatomic location, and were then binarized to become mask images.

For each participant, the mean value across all voxels within the ROI mask was computed as a summary measure of the BOLD response under naproxen and placebo conditions. Values were extracted from first-level 70% MVC COPE images, as we hypothesized that this would induce the highest reported levels of pain intensity. For each ROI hemisphere, summary measure data were analyzed using a paired *t*-test in SPSS version 19. Right and left hemisphere data were analyzed separately as their variances differed between hemispheres. Mean values for each treatment and corresponding standard errors were plotted for ROIs demonstrating significant treatment differences. (A supplementary voxelwise analysis of treatment effects using the 70% MVC class only is provided as part of Supplementary Figure 2, available on the *Arthritis & Rheumatology* web site at http://onlinelibrary.wiley.com/doi/10.1002/art.38987/abstract.)

Pearson product-moment correlation coefficients were computed in SPSS version 19 to assess the relationship between treatment-induced changes in fMRI squeeze task–related VAS pain intensity and treatment-induced changes in mean BOLD responses in selected bilateral ROIs (thalamus, primary and secondary somatosensory cortices, anterior and posterior insula) previously reported to relate to the intensity of perceived pain (29,30). Treatment differences (Δ = naproxen − placebo) were derived for each participant. The significance threshold for all ROI analyses was set at *P* < 0.05. Due to the exploratory nature of these analyses, we did not correct for multiple comparisons. Post hoc sample size calculations to detect significant treatment differences (alpha level of 0.05, 2-tailed, power of 80%) in each ROI in each hemisphere were derived using G*Power version 3.12 (31).

## Results

### Behavioral data (psychometry and pain diaries)

**Figure 2:**
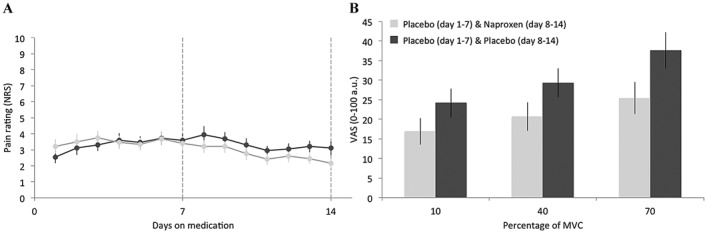
A, Mean ± SEM daily pain intensity determined using a numerical rating scale (NRS) in patients receiving active treatment (light gray; placebo on days 1–7, naproxen on days 8–14) compared to patients receiving placebo (dark gray; placebo on days 1–14). B, Mean ± SEM visual analog scale (VAS) scores during the lateral pinch task. During the scanning visits, participants with painful osteoarthritis of the thumb were cued to perform a lateral pinch (squeeze) at each of 3 different levels (10%, 40%, and 70%) of their maximum voluntary contraction (MVC).

As determined using an NRS, daily pain intensity after 7 days of treatment with naproxen was significantly reduced compared to treatment with placebo (*t*[18] = −3.2, *P* = 0.005), confirming the known analgesic properties of naproxen (Figure 2A). The VAS, present pain intensity (PPI), and sensory subsections of the short form MPQ demonstrated significant reductions after administration of naproxen compared to placebo (VAS rating *t*[18] = −4.34, *P* < 0.001; PPI rating *t*[18] = −3.31, *P* = 0.004; sensory rating *t*[18] = −3.34, *P* = 0.004). No differences were observed for affective ratings on the short form MPQ (*t*[18] = −1.42, *P* = 0.17). Measures of wrist and hand pain and function (the PRWHE) were significantly improved following administration of naproxen compared to placebo (*t*[18] = −2.08, *P* = 0.05). No significant differences between treatments were observed for state anxiety (the STAI-S) (*t*[18] = −1.61, *P* = 0.12) or depression (the BDI-II) (*t*[18] = −0.77, *P* = 0.45), both of which were in the normal range. Psychometric data are summarized in Table1.

**Table 1:** Summary of demographic characteristics and pain and psychometric assessments in the 19 study patients

Variable, session*	Range	Mean ± SD	*t*(18)	*P*†
Age, years	52.00–72.00	60.72 ± 6.44	–	–
Pain duration, years	0.50–17.00	4.03 ± 4.05	–	–
Baseline pain, 0–10			–	–
Screening	2.00–7.00	4.53 ± 1.43		
Familiarization	1.00–8.00	4.21 ± 1.81		
MPQ VAS rating			−4.34	<0.001
Naproxen	9.00–57.00	23.68 ± 11.6		
Placebo	8.00–82.00	39.47 ± 20.12		
MPQ PPI rating			−3.31	0.004
Naproxen	0.00–2.00	0.89 ± 0.65		
Placebo	1.00–2.00	1.52 ± 0.51		
MPQ sensory rating			−3.34	0.004
Naproxen	1.00–17.00	5.73 ± 3.94		
Placebo	3.00–30.00	9.68 ± 7.11		
MPQ affective rating			−1.42	0.17
Naproxen	0.00–3.00	0.21 ± 0.71		
Placebo	0.00–7.00	0.73 ± 1.72		
PRWHE			−2.08	0.05
Familiarization	11.50–68.00	42.39 ± 15.45		
Naproxen	5.50–65.50	28.97 ± 15.08		
Placebo	14.00–73.50	35.92 ± 17.56		
Spielberger STAI state anxiety rating			−1.61	0.12
Naproxen	36.00–80.00	47 ± 11.36		
Placebo	36.00–84.00	50.52 ± 13.14		
BDI-II depression rating			−0.77	0.45
Screening	0.00–23.00	7.05 ± 6.41		
Naproxen	0.00–22.00	6.05 ± 5.93		
Placebo	0.00–26.00	7.05 ± 6.6		

* MPQ = McGill Pain Questionnaire; VAS = visual analog scale; PPI = present pain intensity; PRWHE = Patient-Rated Wrist and Hand Evaluation; STAI = State-Trait Anxiety Inventory; BDI-II = Beck Depression Inventory II.

† Two-tailed.

### In-scanner behavioral data

We sought to investigate differences between naproxen and placebo treatments in in-scanner self-reported pain measures. VAS scores were significantly reduced following administration of naproxen compared to placebo (F[1,18] = 10.61, *P* = 0.004) (Figure 2B). Participants' levels of squeeze (10%, 40%, and 70% MVC) induced a significant increase in VAS scores (Greenhouse-Geisser–corrected F[1.115,20.07] = 12.83, *P* < 0.001) (Figure 2B). There was no interaction between levels of squeeze and treatment. Task performance at 70% MVC evoked the highest perceived pain intensity scores (mean ± SEM VAS score 37.61 ± 4.64 for placebo and 25.379 ± 4.06 for naproxen), providing an appropriate rationale for its use in the ROI analysis.

### Neuroimaging

#### Effects of naproxen on global CBF

Differences between naproxen and placebo conditions in global CBF were not significant (*t*[18] = *−*1.3, *P* = 0.2), suggesting that BOLD signal treatment differences were unlikely to be caused by generalized vascular effects.

#### Whole-brain voxelwise analysis

Whole-brain analyses of task performance fMRI data were performed. Using the average of all levels of load (10%, 40%, and 70% MVC) as an input for each participant in each session, significant reductions in BOLD signal following treatment with naproxen, compared to placebo, were observed in a distributed network of brain regions (see Supplementary Table 1 and Supplementary Figure 2, available on the *Arthritis & Rheumatology* web site at http://onlinelibrary.wiley.com/doi/10.1002/art.38987/abstract). Several of these brain regions were those that we had identified a priori as being important in the cerebral representation of pain. We observed a cluster in the left primary somatosensory cortex and a smaller cluster in the right primary somatosensory cortex. A further large cluster was identified in the thalamus bilaterally that extended anteriorly into bilateral caudate nuclei and the left nucleus accumbens and inferiorly into the left midbrain in an area approximating that of the substantia nigra. Further reductions were observed in the right insula, extending to the temporal lobe, and bilaterally in the amygdalae. We also observed reductions in the right and left frontal lobes and occipital cortices, extending inferiorly into the cerebellum and the posterior aspect of the pons. We did not observe any relative BOLD signal increases following naproxen administration compared to placebo.

#### ROI analysis

**Figure 3:**
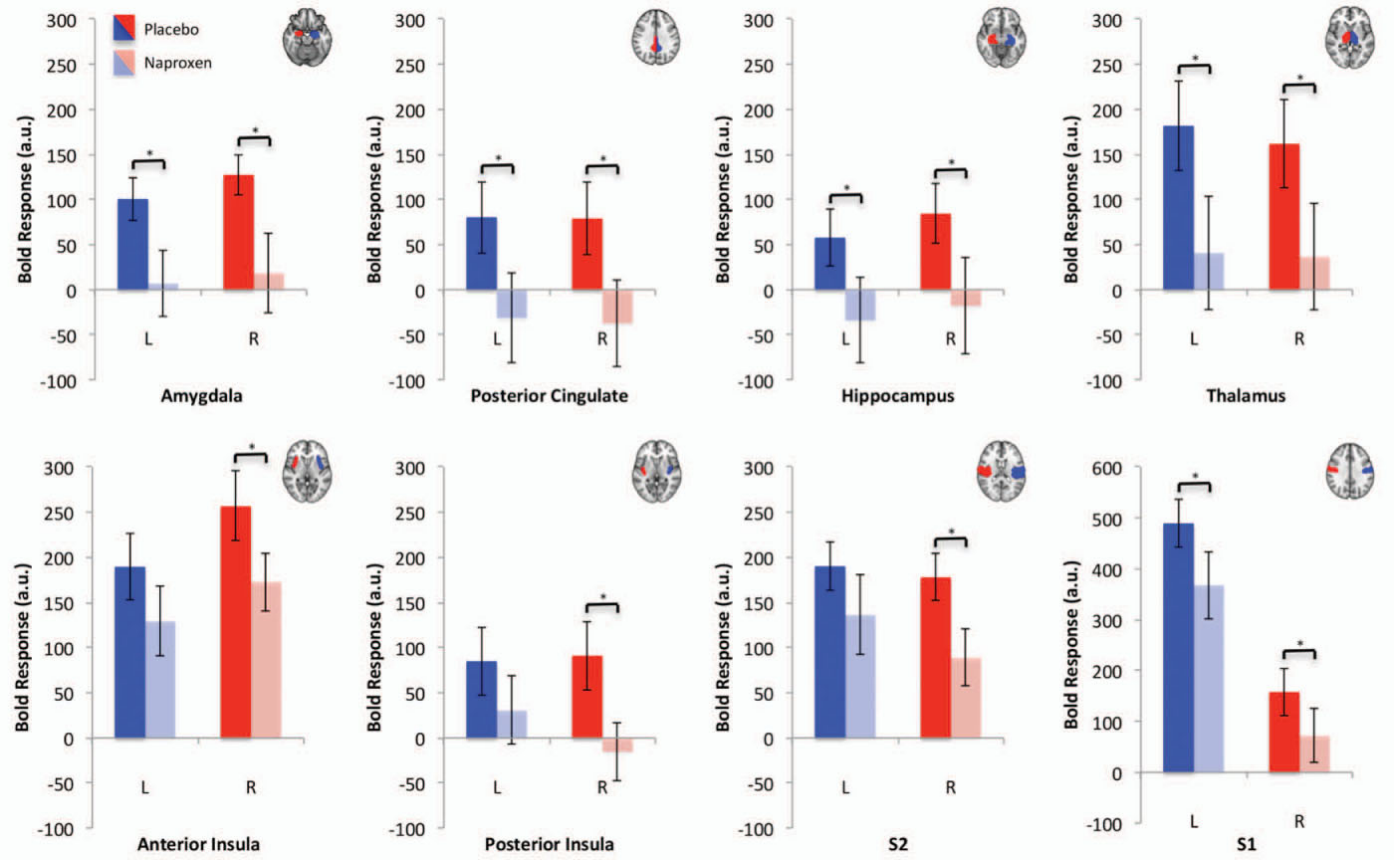
A priori region of interest analysis. Adjacent to each image are corresponding left (L) and right (R) hemisphere mean ± SEM blood oxygen level–dependent (BOLD) responses for patients receiving placebo (bright red/bright blue) and patients receiving naproxen (light red/light blue). ∗ = *P* < 0.05. S2 = secondary somatosensory cortex; S1 = primary somatosensory cortex.

Significant naproxen-mediated reductions in BOLD signal intensity were identified in the amygdala, hippocampal formation, thalamus, primary somatosensory cortex, and posterior cingulate cortex ROIs bilaterally. Further reductions in the right hemisphere only were observed in the anterior and posterior insula cortices and in the secondary somatosensory cortex. Figure 3 illustrates the mean values in significant ROIs at placebo and naproxen sessions. Results of the pairwise *t*-tests for the treatment differences are detailed in Table2.

**Table 2:** A priori ROI analysis*

ROI	Mean response for placebo treatment	Mean response for naproxen treatment	Treatment difference	Standard error of treatment difference	*t*(18)	*P* for *t*(18)†	Pearson's r (n = 19)‡	*P* for Pearson's r†	Sample size§
ACC (L)	132.81	78.08	54.73	43.57	1.256	0.225	–	–	97
ACC (R)	134.38	85.97	48.41	41.75	1.160	0.261	–	–	112
AMY (L)	100.57	6.97	93.60	28.53	3.281	0.004	–	–	16
AMY (R)	127.35	18.40	108.95	33.85	3.219	0.005	–	–	17
HF (L)	57.72	−33.81	91.54	33.65	2.720	0.014	–	–	23
HF (R)	84.44	−17.79	102.23	38.32	2.668	0.016	–	–	23
aINS (L)	189.50	129.33	60.18	39.16	1.537	0.142	0.109	0.657	66
aINS (R)	256.77	172.95	83.82	33.18	2.527	0.021	−0.188	0.440	26
pINS (L)	84.95	30.50	54.45	40.08	1.359	0.191	0.275	0.255	83
pINS (R)	91.15	−15.19	106.34	33.72	3.154	0.005	−0.015	0.952	18
PCC (L)	80.31	−31.21	111.51	49.16	2.268	0.036	–	–	31
PCC (R)	79.04	−37.22	116.26	47.20	2.463	0.024	–	–	27
S1 (L)	489.29	368.04	121.25	55.15	2.198	0.041	0.590	0.008	33
S1 (R)	158.06	72.13	85.93	39.10	2.198	0.041	0.484	0.036	33
S2 (L)	190.13	136.42	53.71	41.09	1.307	0.208	0.646	0.003	90
S2 (R)	177.96	88.88	89.09	33.38	2.668	0.016	0.585	0.008	23
TH (L)	181.76	40.95	140.81	42.21	3.336	0.004	0.460	0.048	16
TH (R)	161.75	36.81	124.94	40.57	3.080	0.006	0.427	0.070	18

* Shown are analyses of the following regions of interest (ROIs) in left (L) and right (R) hemispheres: anterior cingulate cortex (ACC), amygdala (AMY), hippocampal formation (HF), anterior insula (aINS), posterior insula (pINS), posterior cingulate cortex (PCC), primary somatosensory cortex (S1), secondary somatosensory cortex (S2), and thalamus (TH).

† Two-tailed.

‡ Pearson's product-moment correlation coefficient for relationships between treatment-related differences in the magnitude of the blood oxygen level–dependent signal and differences in perceived pain intensity measured by in-scanner visual analog scale recordings.

§ Post hoc sample size calculations (alpha level of 0.05, power of 80%) for each ROI.

**Figure 4:**
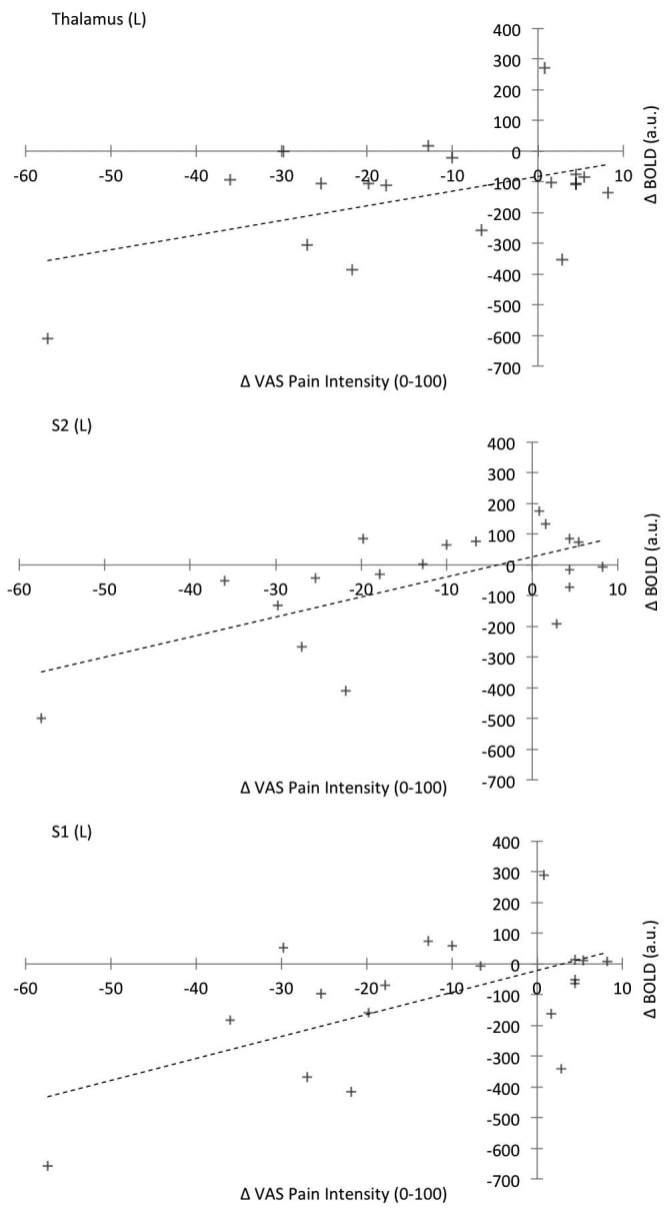
Scatterplots showing the relationship between the change (naproxen condition − placebo condition) in BOLD responses within a particular region of interest (y-axis) and the change in visual analog scale (VAS) scores collected during the task (x-axis). Plus signs represent individual patients. See Figure 3 for other definitions.

Exploring the relationship between treatment-induced changes in task-related VAS pain intensity and mean BOLD responses demonstrated positive correlations in the primary and secondary somatosensory cortices bilaterally and in the left thalamus (contralateral to the affected hand). Representative plots of these relationships (Figure 4) illustrate that reductions in BOLD response following naproxen treatment were associated with reductions in perceived pain intensity. Post hoc calculations of the sample size needed to detect significant treatment effects varied considerably between ROIs, ranging from a minimum of 16 participants (left amygdala and left thalamus) to a maximum of 112 participants (right anterior cingulate cortex) (Table2).

## Discussion

We have demonstrated the sensitivity of BOLD fMRI to detect the effects of naproxen, an analgesic of known efficacy, in a group of individuals with pain secondary to OA of the CMC joint. Local reductions in BOLD signal were identified in brain regions commonly associated with the experience of pain (28), several of which we hypothesized a priori to show changes in response to analgesia. Changes in treatment-related VAS indices of pain intensity correlated with changes in BOLD response in several of these brain regions. These findings demonstrate that BOLD fMRI adds value to conventional self-report measures, offering a mechanistic understanding of both pain and treatment response (11).

Reductions in BOLD response were identified in a distributed network of brain regions following administration of naproxen, compared to placebo. These effects were identified in a real-world sample of patients with painful OA, within the constraints of a fully blinded crossover experimental design. Given the location of reductions in BOLD response following naproxen treatment and the relationship in the thalamus and primary and secondary somatosensory cortices between changes in BOLD signal intensity and perceived pain, we infer that our fMRI findings represent an analgesic effect of naproxen on OA-related evoked pain. The absence of a significant treatment effect on global CBF provides confidence that treatment-derived BOLD signal changes were most likely driven via mechanisms of neurovascular coupling, rather than via a more generalized global increase in CBF (32). We recommend acquisition of imaging data using multiple fMRI modalities, alongside conventional behavioral end points, in order to best understand pain and analgesic responses (11).

Our results build on those of earlier studies of experimental, acute, and persistent pain conditions treated with various analgesic medications, in which treatment effects have been demonstrated by fMRI (e.g., see refs.(33–35)). For the first time, we have demonstrated a robust effect of naproxen on evoked stimulation of pain in the CMC joint with OA. Previous attempts to use pain secondary to OA as a clinical model to assess analgesic effects by fMRI are difficult to interpret, given that these effects were examined in so few patients. The intention of Parks et al (15) to perform a larger fMRI study to evaluate the treatment effects of valdecoxib, a cyclooxygenase 2 inhibitor, was hampered by the withdrawal of this agent from the US market. In a subgroup of only 6 participants, those investigators reported a significant effect of treatment on behavioral indices of background/spontaneous pain but not evoked pain. Interrelationships between drug concentrations, perceived indices of spontaneous pain, and BOLD activity were also described, but caution should be exercised given their derivation from so few participants. While Baliki et al (14) reported treatment effects in their comparison of the effects of lidocaine on chronic low back pain (n = 7) and evoked knee OA pain (n = 5) in the medial prefrontal cortex and thalamus, respectively, the authors acknowledge limitations in that study. Due to small sample sizes, statistical handling of the data limited treatment-related inferences to the study samples rather than to the clinical populations that they represented, and the study had no placebo arm or a control for potential treatment order effects.

Our work adds new knowledge to the field by demonstrating an observable treatment effect in a larger cohort of participants, in a study that was appropriately double blinded and placebo controlled. Post hoc power calculations from this study using a priori–defined ROIs indicated that a minimum of 16 individuals with OA of the CMC joint are needed in order to identify a treatment effect on evoked pain. A sample size of 16 was sufficient only in the thalamus and amygdala contralateral to the affected hand. We included these calculations to guide others in powering future studies, but we recognize that sample sizes may change due to factors including body site, severity of OA pain, paradigm design, and analgesic effect size. It is an unrealistic expectation that treatment effects will modulate brain responses either in all brain regions or in equal magnitude across them. We have shown that changes in brain response relate to perceived pain relief; however, we speculate that the magnitude, direction, and spatial location of these changes are likely to represent a composite generalized “analgesic effect” and response characteristics unique to the analgesic under assessment. In light of increasing evidence of the impact of persistent pain states on both brain structure and function (36,37), use of anatomically defined ROIs, while unbiased (38), may also partially account for variability in required sample sizes across ROIs. However, given the broad acknowledgment that pain is a multifaceted experience underpinned by a complex network of brain regions (39), it is of course overly simplistic to ascertain treatment effects from single ROIs in isolation.

A critical quality provided by the use of neuroimaging end points is the value that they add to conventional behavioral indices, providing mechanistic correlates of persistent pain (24) and treatment effects. Previous studies (15,40) have shown perturbed affective responses to pain in OA; these have recently been theorized to be maladaptive assessments of and responses to pain as a threat to homeostatic body functioning (24). Subtle variations in the location of supraspinal pain responses in these studies may reflect heterogeneity in clinical pain states, but together they add weight to a developing view that persistent OA pain may be, at least in part, maintained by changes in central as well as peripheral nervous system functioning (14,15,24,40). Neuroimaging reports have also described altered functioning of endogenous pain control mechanisms in individuals with painful OA. A recent fMRI study (5) showed the presence of neuropathic-like symptoms in patients with OA, using the PainDETECT questionnaire (41). When fMRI was combined with quantitative sensory testing, hyperalgesic responses to punctate stimuli were associated with significant periaqueductal gray matter activity, leading the authors to interpret that the periaqueductal gray matter has a role in central sensitization in at least some patients with painful hip OA. Similarly, our own work examining background pain in individuals with painful hand OA, compared to healthy controls, showed differential CBF between groups in the periaqueductal gray matter and other descending modulatory systems in the midbrain and brainstem (24).

These findings provide important insights for interventions aimed at the reduction of OA-related pain, which currently remain focused on reducing or eliminating potential peripheral generators of nociception, for example, joint replacement surgery (42,43). Outcomes of these interventions are not always satisfactory for individuals (for review, see ref.(44)), which may be due in part to supraspinal pain mechanisms remaining essentially uncharacterized and untreated. In this work, we did not find a modulatory effect of naproxen in these regions. Type II errors aside, these null findings suggest the working hypothesis that the analgesic effect of naproxen does not modulate descending pain control system responses to evoked pain. Specifically designed new studies will be needed to test this.

In this study, we combined a “gold standard” pharmacologic MRI analgesic design methodology with a novel functional task and a mock scan protocol. This design aims to account for additional influences on BOLD response, including treatment order, task demands, anxiety, mood, and placebo responses. Psychometric data on anxiety and depression were stable across sessions, reducing the likelihood that they influenced the fMRI results. Our chosen task differs from conventional evoked-response paradigms in that pain responses are elicited with an accompanying motor component. However, in light of recent comments that pain may represent an actual or perceived threat to the body (45,46), we speculate that experimental paradigms that promote genuine pain-provoking actions, rather than those that elicit responses to on/off noxious stimulation, may represent a more comprehensive cerebral fingerprint of pain response in OA (47).

In summary, our study demonstrates the sensitivity of BOLD fMRI to detect the mechanisms underlying treatments of known efficacy in OA. The locations of the effects were specific and physiologically plausible, and treatment-related changes in VAS scores were related to changes in BOLD response in brain regions underpinning sensory discriminative aspects of the pain experience. These results demonstrate the enticing potential of fMRI as an adjunct to self-report for detection of early signals of efficacy of novel pharmacologic and nonpharmacologic treatments, in small numbers of individuals with persistent pain (see refs.(12) and(48)), which in turn may provide them with reduced pain and increased quality of life.

## Author Contributions

All authors were involved in drafting the article or revising it critically for important intellectual content, and all authors approved the final version to be published. Dr. Howard had full access to all of the data in the study and takes responsibility for the integrity of the data and the accuracy of the data analysis.

**Study conception and design.** Sanders, Huggins, Vennart, Massat, Williams, Howard.

**Acquisition of data.** Sanders, Krause, Choy, Williams, Howard.

**Analysis and interpretation of data.** Sanders, Krause, O'Muircheartaigh, Thacker, Huggins, Vennart, Massat, Choy, Howard.

## Role of the Study Sponsor

This project was an academic–industrial collaboration between King's College London and the study sponsor, Pfizer Global Research and Development, UK. Pfizer and King's College London scientists worked in collaboration on the following areas: study design, data analysis, interpretation of data, and the writing of the manuscript. All data collection was performed by King's College London scientists only. Pfizer approved the content of the manuscript prior to submission.
