# Supplementary material for: Pharmacologic Modulation of Hand Pain in Osteoarthritis: A Double-Blind Placebo-Controlled Functional Magnetic Resonance Imaging Study Using Naproxen
Source: Arthritis Rheumatol. 2015 Feb 25;67(3):741–51. doi: 10.1002/art.38987 (PMC4365729; doi:10.1002/art.38987)
Supplement: Supplementary file 1 [file art0067-0741-sd1.doc]

**SUPPLEMENTARY MATERIALS**

**Inclusion Criteria**

Participants were required to meet all of the following inclusion criteria to be eligible for enrollment into the study:

1. Right-handed, post-menopausal female, any race, 50-80 years.
2. Body Mass Index (BMI) of less than approximately 36 kg/m^2, body mass less than 110 kg and width at shoulders and hips less than 50 cm.
3. Dominant right-handedness trait (ie, the participant writes with her right hand).
4. The clinical diagnosis of osteoarthritis of the hand, according to American College of Rheumatology guidelines, of at least 6 months duration.
5. Pain around one of the first CMC joints due to the osteoarthritis. The participant must have reported the estimated average pain in this joint in the week prior to questioning as at least 4 on an 11- point numerical rating scale at the time of screening or at randomization.
6. Pain elicited in the one of the first CMC joints by a task requiring the participant to grip a key- like device at the screening visit.
7. Evidence of a personally signed and dated informed consent document indicating that the participant (or a legally acceptable representative) has been informed of all pertinent aspects of the trial.
8. Willingness and ability to comply with scheduled visits and trial procedures.

**Exclusion Criteria**

Participants with any of the following were excluded from the study:

1. Participants who consumed more than 8 cups of caffeinated drinks per day (1 caffeinated drink only was permitted early in the morning of the scanning visits).
2. Any participants who smoked more than 5 cigarettes per day (no smoking was permitted on the scanning visit days prior to scanning or whilst in the centre).
3. Participants with a history of any major psychiatric disease.
4. Participants with known or a history of alcoholism or hepatic cirrhosis, or with symptoms or history of congestive heart failure, or uncontrolled hypertension, or established ischemic heart disease. Participants with any psychological disease that required ongoing psychoactive drugs including tricyclic antidepressants or dopamine and/or serotonin reuptake inhibitors were excluded unless the dose had been stable for at least 3 months and was not anticipated to change.
5. Participants unable to lie still within the environment of the MRI scanner for the period required to perform the study and those where MRI scanning was contraindicated (claustrophobia, metal implants, pacemaker, etc).
6. Participants with serious hepatic, respiratory, neurologic (epilepsy, multiple sclerosis), hematologic or immunologic illnesses, an unstable cardiovascular disease, or any other severe acute or chronic medical or psychiatric condition or laboratory abnormality that may have increased the risk associated with trial participation or may have interfered with the interpretation of trial results in the judgment of the investigator.
7. Participants with a basal creatinine clearance <20 mL/min.
8. Participants receiving any anticoagulants, with exception of Aspirin use ≤125 mg per day for cardiovascular prophylaxis.
9. Participants with active malignancy of any type or a history of a malignancy (with the exception of Participants with malignancy surgically removed with no evidence of recurrence within 2 years before enrolment and Participants with a history of treated basal cell carcinoma).
10. Participants who have previously been administered naproxen at doses of at least 1 g total daily dose for at least 7 days and who failed to experience pain relief, or those who have previously been unable to tolerate naproxen or its incipients for any reason.
11. Participants experiencing other severe pain which, in the opinion of the investigator, may impair the assessment of the pain due to osteoarthritis.
12. Participants with any skin condition over the CMC joint that will be used for pain threshold testing, which may interfere with the assessment of pain thresholds.
13. Participants who have experienced acute joint trauma of the hand within 12 months of commencing the study.
14. Participants who have used any of the prohibited medications as listed below:
    1. Oral or intramuscular corticosteroids within 4 weeks prior to randomisation.
    2. Monoamine oxidase inhibitors within 2 weeks prior to randomisation.
    3. Analgesic agents, other than NSAIDs, NSAID/codeine, Cox-2 inhibitors, paracetamol or paracetamol/codeine within 1 week prior to randomisation.
    4. Intra-articular steroids into the study joint within 12 weeks, and to any other joint within 4 weeks prior to randomisation. Participants with any gastrointestinal issues that in the opinion of the investigator would have interfered with the absorption of study medication.
15. Participants who have difficulty swallowing tablets or are unable to tolerate oral medication.
16. Participants with a medical history of stomach or duodenal ulceration or gastroesophageal reflux disease.
17. Participants with a history of NSAID-induced asthma, rhinitis, nasal polyps or urticaria.
18. Participants who have participated in a clinical trial for an investigational drug within 90 days of screening.

**Randomization Criteria**

Participants were randomized into the trial provided they satisfied all selection criteria and the following by the completion of the down-titration of existing applicable medications (see below)

• The participant must have reported an estimated average pain score of at least 4 on an 11-point numerical rating scale in one of the first CMC joints in the week prior to randomization if this was less than 5 at screening.

**Life Style Guidelines**

**Down-titration Of Existing Analgesic Medication**

Where possible, participants medications were down-titrated in a manner that allows approximately 7 days free from their analgesic therapy (except paracetamol and paracetamol/codeine preparations) prior to the familiarization/randomization visit.

Chronically dosed NSAIDs (including codeine, if appropriate) and Cox-2 inhibitors were required to be washed out for 14 days.

**Meals and Dietary Restrictions**

Consumption of a light breakfast was permitted on the morning of each scanning session. Lunch was provided by the site.

**Alcohol, Caffeine and Tobacco**

Participants abstained from alcohol for 24 hours prior to each scanning session.

A single caffeinated drink was allowed on the morning of the scanning session. Following this, all caffeine-containing products were prohibited until completion of each scanning session.

Participants were required to abstain from the use of tobacco- or nicotine-containing products for 4 hours prior to admission until discharge for each scanning session.

**Activity**

Participants were asked not to alter their current exercise regimen or the types of activities that they would normally carry out for the duration of the study as this may influence their pain scores.

**STUDY TREATMENTS**

Participants who met the eligibility criteria were randomized in a 1:1 ratio to one of two sequences (Section 5.1) and began their placebo run-in period during which placebo was administered 14 times (BID). This period was followed by blinded treatment with naproxen or matching placebo tablets for another 14 doses. Participants were administered naproxen 500 mg BID, beginning on the evening of Day 1 or Day 15 and completed treatment in the morning of Day 8 or 22. Those participants randomized not to receive naproxen for either of these periods, received matched placebo BID instead.

A 1-week interval during which placebo BID was administered served as a washout between Periods 1 and 2; this period also served as a placebo run-in for Period 2. All groups were blinded throughout study treatment (placebo run-in, treatment and washout).

Treatments were as follows:

- Naproxen tablets, 1 g total daily dose (500 mg BID).
- Matching placebo tablets (BID).
- Treatment periods, each of which included placebo run-in and fixed dosing were as follows:
- Period 1: Days -7 to 8.
- Period 2: Days 8 to 22 (equivalent to days -7 to 8 of Period 1).

**Allocation to Treatment**

Each participant was assigned to one of the following sequences of treatment administrations by means of a computer-generated, pseudo random code. The sequences used were:

| Sequence | Period 1 | Period 2 |
| --- | --- | --- |
| I | A | B |
| II | B | A |

where the treatments to be administered were:

A:  Naproxen B:  Placebo

The Investigator assigned randomization numbers (participant numbers) 1 to 24 to the participants at the familiarization/randomization visit as they were determined to be eligible for randomization. A statistician had access to the randomization. If a participants was withdrawn from the study and a replacement required the replacement participant was allocated to the same treatment sequence as the participant they replaced.

**Drug Supplies**

Drugs were supplied by Pfizer Ltd.

**Administration**

Instructions were provided to participants to take one tablet in the morning and evening (approximately 12 hours apart) with or after food. Each tablet was taken with a glass of water.

**MRI Scanning Parameters**

*EPI:* number of slices=42, slice thickness=3mm, inter-slice gap=0.33mm, field of view (FOV)=240x240mm, acquisition matrix=64x64, repetition time (TR)=3000ms, echo time (TE)=30ms, and flip angle=90^o^.

SPGR: number of slices=196, slice thickness=1.1mm, inter-slice gap=1.1, FOV=280x280mm, acquisition matrix=256x256, TR=7.008ms, TE=2.8ms, inversion time=450ms, and flip angle=20^o^

PCASL: PCASL acquisition and CBF computation are described in detail here: [**http://www.kcl.ac.uk/iop/depts/neuroimaging/research/pain/pCASLdetail.pdf**](http://www.kcl.ac.uk/iop/depts/neuroimaging/research/pain/pCASLdetail.pdf)

Supplementary Figure 1. A) Schematic of choice of three targets (10, 40 or 70% of individualised MVC) presented to participants during fMRI; B) Single trial presentation as viewed by participants. Force is applied to squeeze device until feedback bar (in red) passes target arrow. Target disappears when force applied is equal to or greater to the target for one second; C) Image of MRI-compatible squeeze device D) Projected image MRI-compatible VAS controlled by participants’ left hand.

Supplementary Table 1. Brain regions demonstrating significantly reduced BOLD response following naproxen, compared to placebo administration. Location of peak values of the Z-distribution, in MNI co-ordinate space, are reported for significant (p<0.05, cluster-corrected) local cluster maxima.


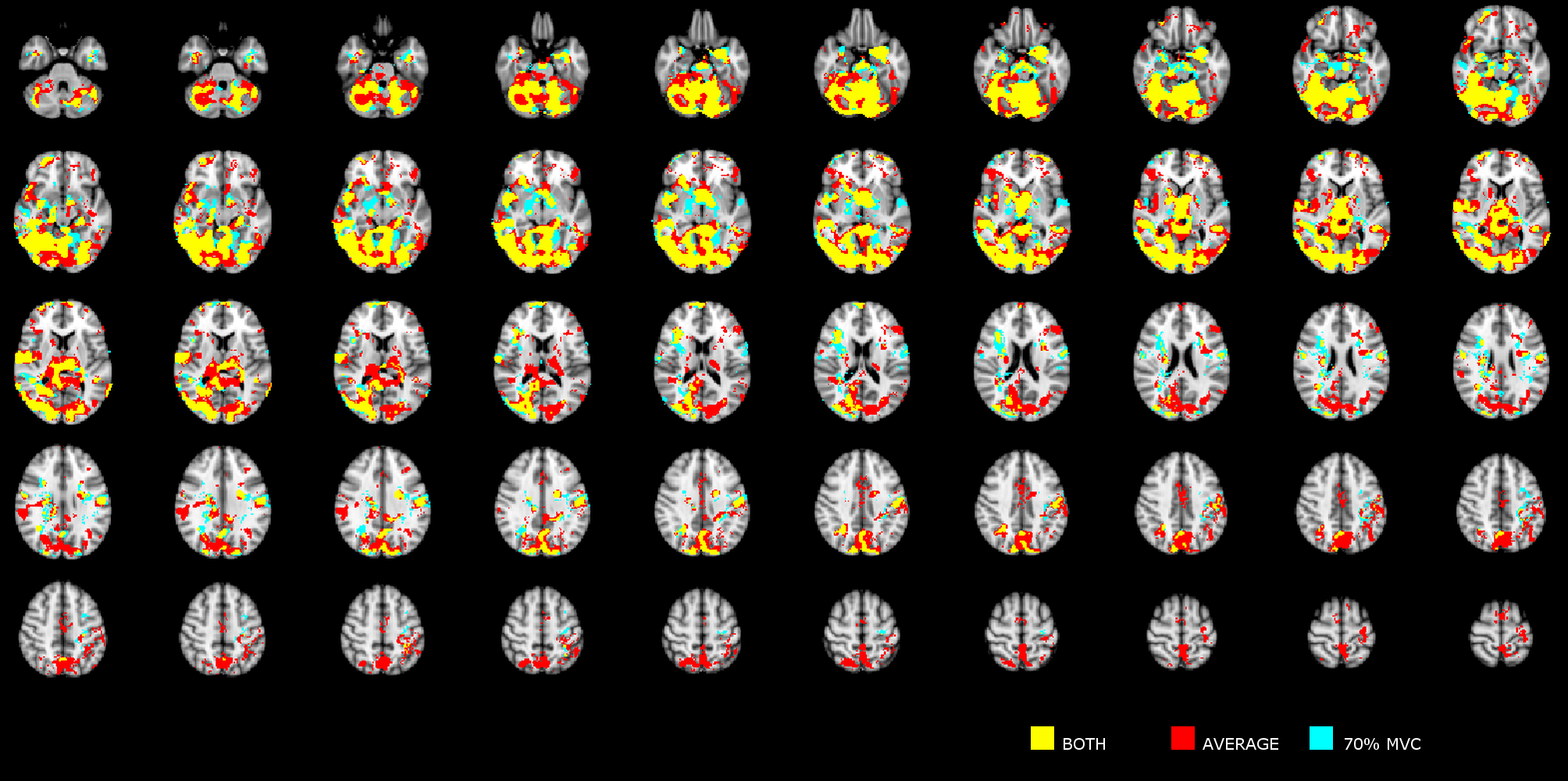


Supplementary Figure 2. Lightbox array illustrating significant (p<0.05, cluster-corrected) voxelwise decreases in brain activation following naproxen, compared to placebo administration. Cluster masks for average 10-40-70% MVC analysis are illustrated in red, 70% MVC only analysis in cyan. Significant regions common to both analyses are highlighted in yelllow.
